# Better RED than dead: paying the people for environmental services in Amazonia

**DOI:** 10.1098/rstb.2007.0034

**Published:** 2008-02-11

**Authors:** Anthony Hall

**Affiliations:** Department of Social Policy, London School of Economics and Political ScienceHoughton Street, London WC2A 2AE, UK

**Keywords:** payments for environmental services, reduced emissions from deforestation, *Proambiente*, Amazon, small farmers

## Abstract

The introduction of payments for environmental services (PES) offers an opportunity for traditional and indigenous populations to be compensated for contributing to carbon sequestration in meeting the challenge of ameliorating global warming. As one mechanism among several for promoting biodiversity conservation and sustainable development, pro-poor PES initiatives could eventually be incorporated into an international post-Koyoto framework to encourage reduced emissions from deforestation. Brazil's *Proambiente* PES scheme for small farmers in Amazonia has enjoyed some limited success, but it has fallen short of expectations. Its performance has been undermined by the lack of a national legal framework, limited funding, reduced implementation capacity, poor cross-sector collaboration and incompatibility with existing regional development policies. These challenges are being addressed by the federal government in cooperation with civil society with a view to scaling up *Proambiente* into a national programme.

## 1. Introduction

The growing pace of Amazon deforestation has become a key issue in the debate on climate change. Globally, land use changes including forest loss account for approximately 18–20% of total greenhouse gas (GHG) emissions. Yet Amazonian deforestation is responsible for three-quarters of GHG emissions in Brazil, which is itself the world's fourth largest emitter after China, the US and Russia. Since the 1960s, the Brazilian Amazon has been treated as a virtually free resource to be exploited in the name of national development with little or no regard for the huge environmental and social costs involved. With some 20% of Brazil's Amazon rainforest now lost to clear-felling, over half of the region has been adversely impacted by human activity. It has been estimated that up to 50% of the country's rainforest could disappear by 2050, generating a regional average temperature rise of up to 4°C and a rainfall reduction as high as 20%, with disastrous consequences including widespread ‘savannization’ and forest die-back (Betts *et al*. 2008; Greenpeace 2006; INPE 2007; Nepstad *et al*. 2008; Sawyer 2008).

Successive military and civilian governments have since the 1960s encouraged settlement through cattle ranching, logging, soybean cultivation and occupation by small farmers. Historically, producers in the Amazon have been actively encouraged to remove the rainforest as proof of ‘productive’ activity under land-titling laws and for the acquisition of credit. Environmental policy has been based on conservation in protected areas backed up by punitive, command-and-control measures. These are necessary but of limited effectiveness on their own in fighting increasing rates of deforestation in the region and the consequent loss of environmental services such as carbon sequestration, biodiversity preservation and watershed management.

A major challenge facing policy-makers today is thus how to move away from the current system of perverse incentives which stimulates deforestation, and towards policies that encourage forest preservation along with more sustainable forms of land settlement and production. The present paper examines Brazil's very embryonic efforts at persuading small farmers to adopt such practices by paying them for environmental services rendered, encouraging them to limit forest removal, reduce destructive activities such as continuous slash-and-burn farming and set up more sedentary, ecologically friendly production systems such as agroforestry and extractivism.

After reviewing Amazon settlement policies and the potential importance of providing payments for environmental services (PES) to small farmers, the experience of Brazil's pro-poor *Proambiente* PES programme will be examined.^1^ It will be argued that, despite being fraught with problems, *Proambiente* is one tool among many which could reward small producers for enhancing carbon sequestration and biodiversity conservation. If an international post-Kyoto framework for encouraging reduced emissions from deforestation (RED) beyond 2012 is negotiated under the auspices of the United Nations Framework Convention on Climate Change (UNFCCC), funds for this purpose could be channelled from the North to the South through official aid and/or carbon trading.

## 2. Peopling the Amazon

For as long as the development of Amazonia has been discussed in scientific and policy circles, the role of its traditional and indigenous populations in moulding the region's past and its future has been marginalized. This was most apparent during the early days of the modern settlement era in the 1970s, when the Amazon was treated as a demographic void by Brazil's military rulers who urged the allocation of ‘land without men to men without land’ (Hall 1989). This conveniently ignored the presence of pre-existing populations as the generals pursued a number of diverse and often conflicting goals. These ranged from national integration and economic modernization to poverty alleviation and social stabilization in other regions of the country through the export of ‘surplus’ people to the ‘empty’ frontier.

For three decades, strong official backing was given to large landowners and investors in key sectors such as livestock, logging and mining, which monopolized government subsidies. Small settlers, in contrast, were drawn to the region but then usually denied significant support and largely abandoned to their fate. Furthermore, in an exercise of ‘blaming the victims’, they were often portrayed by the politically powerful as destroyers of the environment who made little positive contribution to regional progress (Bourne 1978; Schmink & Wood 1978; Branford & Glock 1985; Mahar 1988; Hecht & Cockburn 1989). For many years, the word *povo* (the ‘people’) was literally, in the eyes of most politicians and official policy-makers, a four-letter word, the very antithesis of development.

This perception was challenged in the late 1980s and 1990s by the people themselves. Amazonia's social movements—the rubber tappers, small producers and indigenous groups—vociferously reminded the world that they too played a key role in using and conserving natural resources while promoting economic development and serving national interests (Hall 1997). In protest at growing deforestation and abuses such as the murder of rubber tappers' leader Francisco ‘Chico’ Mendes in 1988, the international environmental lobby, foreign aid agencies and global public opinion generally aligned with domestic groups to pursue a new policy agenda highlighting the contribution of local populations to the sustainable development of Amazonia. This came at a propitious moment in the wake of the Brundtland Report (1987), followed by the Earth Summit (1992) held in Rio de Janeiro, a combination of events which sparked significant environmental policy changes and institutional reorganization in Brazil under Presidents Fernando Collor de Mello and José Sarney (Hall 2000).

Yet despite this recent trend, mainstream environmental policy in the Amazon has been dominated by a command-and-control approach based on conservation together with the application of punitive measures against those found guilty of breaking the law. Implementation is notoriously difficult in an area a huge as Amazonia. In 2005 Brazil's environmental control agency IBAMA allocated 850 officials to a region of 1.9 million square miles, one staff member for every 2300 square miles on average. Operations are generally under-funded and only a very small proportion of fines is ever collected.^2^

Approximately 40% of Brazilian Amazonia is currently set aside in protected areas for both total conservation and sustainable use, including indigenous reserves. Over 60% of this area involves the direct participation of resource-user populations in managing these units. A range of projects and programmes both within and outside of these protected areas has been developed to directly benefit local groups such as extractivists, small farmers, fishing communities and indigenous groups; and producers of various kinds whose livelihoods depend upon the non-destructive use of natural resources but which also contribute to local economic development (Hall 1997). Approximately 20% of Brazilian Amazonia's current population of some 20 million, or approximately 4 million rural inhabitants, still depend for their economic survival on the region's natural resources. These include, for example, extraction of tree fruits and other crops, agroforestry systems, community-based logging, sustainable fishing and pasture management.

Yet despite significant progress in this area, the vast majority of small farmer settlers in the Amazon still practise traditional slash-and-burn farming, which contributes to growing deforestation and environmental degradation. It is estimated that they are responsible for approximately 20% of deforestation in Brazilian Amazonia overall. Cattle ranching and illegal logging (as well as soybean indirectly) account for approximately 70% and mining for the remainder (Fearnside 2005). By incorporating traditionally marginalized small farmers into sustainable development initiatives such as PES, the loss of forest cover and associated environmental services could be ameliorated.

## 3. PES and RED

Environmental or ecosystem services ‘are the conditions and processes through which natural ecosystems, and the species that make them up, sustain and fulfil human life’ (Daily 1997, p. 3). Specific environmental services associated with preservation and agricultural diversification include carbon sequestration, watershed management, biodiversity conservation and the preservation of landscape beauty. To qualify for PES, services rendered should ideally be measurable; for example in terms of tons of carbon captured, biodiversity preserved, deforestation avoided or volume of clean water supplied. PES projects should be monitored for compliance and their performance certified before payments are made to service providers (Wunder 2005).

Official subsidies for Amazon settlement have historically encouraged deforestation rather than conservation.^3^ Yet PES could help alter this perverse pattern. One such set of policies involves providing financial rewards to rural landowners and resource-users who adopt environmentally friendly practices, ranging from outright conservation to sustainable development techniques. Financial compensation in the form of PES rendered would reward resource-users for their efforts to either preserve forests and other natural resources intact, and/or introduce production systems that generate economic surplus and sustain local populations without destroying the resource base upon which people's livelihoods depend.

Measures include the conservation of forested areas designated as ‘legal reserves’ under Brazil's Forest Code (1965), replanting of forests, introduction of agroforestry systems, extraction of forest products such as rubber, fruits and nuts, reduction or elimination of fire-use in land preparation, pasture management and so on. PES schemes offer the potential for channelling financial rewards to groups of small landowners and resource users whose environmental contribution would otherwise remain unrecognized by mainstream government policy, which generally favours large-scale, export-oriented commercial producers.

Policy initiatives to reduce the rate of tropical deforestation are especially relevant in Brazil where forest loss is responsible for three-quarters of national GHG emissions and contributes significantly to global warming (Stern 2006). This discussion is also poignant in the light of pressures by some developing countries to introduce the notion of ‘avoided deforestation’ as a valid criterion for carbon offsetting. This is currently not permitted under the Clean Development Mechanism (CDM) during the first phase of the Kyoto Protocol (2008–2012), which allows offsets to be generated only through reforestation and afforestation projects. However, the issue of reduced emissions from deforestation (RED) is under consideration by the scientific body of the UNFCCC. This refers to the maintenance of standing forest in order to reduce GHG emissions (Ebeling & Yasué 2008; Lemos & Roberts 2008).

Pressure is mounting to include incentives for RED during the second phase of the Kyoto Protocol after 2012. The World Bank is leading this initiative and is seeking approval from the G8 for a ‘Global Forest Alliance’ (GFA) which would bring together major conservation NGOs and the private sector. Within the GFA, a $300 million Forest Carbon Partnership Facility (FCPF) would pilot test schemes for reducing emissions from deforestation. Even Brazil, which for a variety of reasons has long resisted such pressures (Fearnside 2001), has warmed somewhat to the idea. Although it currently rejects the market-based approach suggested by the Coalition of Rainforest Nations (CRN 2007),^4^ Brazil has proposed instead a ‘compensated reduction’ facility to pay countries that reduce rates of deforestation from an international donor fund (Santilli *et al*. 2005; Lemos & Roberts 2008).

## 4. PES and *Proambiente*

This section will consider the emergence of PES policies in Brazil and the conception of *Proambiente* as a mechanism for benefiting small farmers in the Amazon. It examines the structure and initial results of the scheme in compensating small producers, together with some implications for future PES policy in Brazil, before considering wider issues under proposed RED initiatives.

### (a) The PES principle

PES in Brazil have so far been delivered largely through industrial carbon sequestration initiatives under the CDM such as landfill projects as well as a handful of forestry schemes mainly outside of the Amazon.^5^ Yet the potential for including small farmers and other resource users in certain key areas of Amazonia as part of a future RED strategy for emissions reductions policy could be significant. This would serve not just as one vital element of environmental action, offering financial incentives to enhance conservation and development efforts. It would also strengthen people's livelihoods and help support the activities of those grassroots groups that act as custodians of the forest and suppliers of key services. Research elsewhere has shown that ‘direct payments benefit poor farmers by improving cash flows, providing a fungible store of wealth, and diversifying sources of household income’ (Ferraro & Kiss 2002, p. 1718).

In Brazil, the potential of such direct payments for promoting conservation and development was realized early on. In 2000 rural unions, environmental NGOs and community groups in Brazil's Amazon region jointly conceived a ‘Programme for the Socio-Environmental Development of Rural Family Production’ (*Proambiente*), following lengthy debates over the future of small-scale production in Amazonia.^6^ This would reward small farmers and other producers for providing environmental services in 12 key areas or ‘poles’ distributed over several Amazon states.^7^ With the election in late 2002 of Luiz Inácio Lula da Silva, Brazil's first working class president, and a PT government seen as favourable to grassroots interests, the project was transferred from civil society to the Ministry of the Environment from 1 January, 2004.

*Proambiente* is ‘…directed at rural family production, agro-forestry, extractivism, fishing, indigenous and other forms of traditional production.’ Under the scheme, such groups ‘…would cease to be regarded merely as suppliers of primary produce but be valued for their multi-functional contributions to economic production, social inclusion and preservation of the environment… (facilitating)… compensation for environmental services rendered to Brazil and the world’ (Proambiente 2003, pp. 2–6). Specific environmental services in this context were defined as: (i) reduction or avoidance of deforestation; (ii) carbon sequestration; (iii) recuperation of ecosystem hydrological functions; (iv) soil conservation; (v) preservation of biodiversity; and (vi) reduction of forest fire risks.

Avoided deforestation and carbon sequestration would be monitored through direct indicators, and the remaining impacts via indirect measures inherent in the programme's certification process (Proambiente 2003). In this way, it was expected that reductions in forest loss and the amount of carbon thus captured by changes in land use against an established baseline could be monitored for each development ‘pole’. The process followed a number of fixed stages: (i) preparation of sustainable development plans for communities (*Planos de Desenvolvimento Sustentável*), (ii) drawing up of resource utilization plans (*Planos de Utilização*), (ii) negotiation of community agreements (*Acordos Comunitários*), (iii) auditing of activities for their rendering of environmental services (*Auditorias de Campo*), (iv) certification of activities (*Cédula de Certificação de Serviços Ambientais*), and (v) disbursement of payments.

Independent monitoring would be undertaken using a combination of satellite imagery, mapping and field checks on the ground. Assuming a favourable outcome, participating households would be paid the equivalent of half a minimum salary per month (US$95) to reward the provision of environmental services. This verification process would be repeated on an annual basis to justify the continuation of such payments. Shortfalls in the provision of environmental services by participants would result in reduced monthly payments on a sliding scale.

*Proambiente* is conceived in principle as a cross-sector initiative involving various ministries although, as noted below, such collaboration has proved problematic. Led by the Ministry of the Environment (MMA) under the Secretariat for Extractivism and Sustainable Development, agricultural extension support is provided through the Ministry of Agrarian Development (MDA) and its Secretariat for Family Agriculture. In the original plan, subsidized credit for small producers (PRONAF) was to be made available via the MDA and the Bank of Brazil, although this has not materialized due to a series of problems mentioned below. Certification would be the responsibility of the official agricultural research organization EMBRAPA and other agencies, while monitoring of carbon stocks would be undertaken by the Ministry of Science and Technology (MCT), EMBRAPA and the civil household (casa civil) of the Presidency.

A Socio-Environmental Fund (*Fundo Socioambiental*) to be administered by the MMA was conceived to finance payments to farmers, bringing together fixed sources from government and variable contributions from international donors and private companies, including carbon offset purchases (Proambiente 2003). Following successful piloting of *Proambiente*, it was hoped that the PES principle would be substantially scaled up and applied as a national policy.

### (b) Results and challenges for Proambiente

After 4 years under implementation, *Proambiente* has had mixed results. Of the 12 original ‘poles’, 10 have become operational with some 4200 participating families, of whom a total of 1768 (42%) have received total payments averaging R$650 (US$325) per household (Viana *et al*. 2006). For legal reasons it has not so far been possible to set up a permanent fund as originally envisaged. Thus, it was necessary to seek ‘emergency’ support through the Demonstration Projects component of the G7 Pilot Programme, which has historically supported *Proambiente*-type activities in agroforestry, extractivism and related fields. Yet this was a stop-gap solution that could not do justice to the project's original financial objectives in paying farmers. This is, not unsurprisingly, regarded by *Proambiente* farmers very much as a token payment that fell far short of what had been promised.

Prophetically, an official assessment of the prospects for *Proambiente* made shortly after the project's handover to government in 2004 had already predicted that a shortage of funding and support capacity would enable less than half of the programme's target number of families to be reached beyond the 2-year initial phase (MMA 2004). Despite the limited scale and effectively ‘pilot’ nature of *Proambiente*, however, the government hopes to use it as a model for the introduction of a national PES programme (Viana *et al*. 2006). Yet before this is even remotely possible, a number of major financial, legal, political and bureaucratic challenges have to be addressed.

The first hurdle to be overcome is the lack of legal recognition in Brazil at federal level for the concept of environmental services and their economic value. Though Brazilian legislation makes provision for water-use charges, for example, it does not place an economic value on the water-conservation role of landowners. Brazil has a number of instruments that provide PES, but it has no national PES policy. Closely allied to this is a second major obstacle, the absence of a permanent source of public funding drawn from taxation and compensatory economic instruments to guarantee some financial continuity for *Proambiente*.

Brazil's first such legislation has recently been enacted by the state government of Amazonas, which in June 2007 introduced a ‘Law on climatic change, environmental conservation and sustainable development’^8^ (Amazonas 2007; Radiobrás 2007). A ‘Sustainable Amazonas Foundation’ has been set up with a trust fund of R$40 million (US$20 million). Half is provided by the state government and half by the Bradesco bank. Further funds will be raised from the sale of carbon credits for avoided deforestation in the voluntary market, generating up to an estimated US$100 million a year (FOE 2007). Under the scheme, a monthly ‘forest grant’ (*Bolsa Floresta*) of R$50 (US$25) will be paid to over 4000 households in five ‘sustainable development’ protected areas, extending to 8500 families by the end of 2008. The aim is to support traditional populations in their pursuit of non-destructive activities such as extractivism, fishing and tree fruit cultivation and to discourage illegal deforestation. In future, Brazil's states could bear a primary responsibility for forest management and introduction of PES schemes.^9^

Federal legalization for PES is being addressed by the national government (in late 2007) through draft bills, two of which have been presented in the Chamber of Deputies, and a third is to be added.^10^ When and if approved by Congress, this new legislation would establish the concept of PES in Brazil and set up a National Programme of PES or ‘Green Fund’ (*Bolsa Verde*) for small farmers to be financed by international donations. A third bill is to be introduced by the Executive which would attempt to secure more permanent funding from the national budget and a variety of other domestic as well as international sources.^11^ Together, these three bills could provide a legal and financial basis for expanding *Proambiente* into a national programme. It has been estimated that a large-scale PES programme involving 10% of Brazilian Amazonia's small farmer population in degraded areas would cost R$90 (US$45) million a year to conserve 3.75 Mha—approximately one-tenth of the rainforest (Viana *et al*. 2006). However, it would also be necessary to increase core government funding for the MMA, whose implementation capacity has been undermined by inadequate allocations and by spending cutbacks, suggesting a low official prioritization of environmental policy (Dutra *et al*. 2006; Arruda *et al*. 2007).

A third set of issues concerns the compatibility of *Proambiente* with other government policies for small producers and the extent of cooperation among relevant ministries and implementing agencies.^12^ For example, small-scale rural production is the responsibility of the Ministry of Agrarian Development (MDA) which uses diverse lines of agricultural credit through its National Programme for Strengthening Family Farming (PRONAF). However, the bulk of this funding (85%) is geared towards investments and running costs for low-risk borrowers regarded favourably by bank managers. These categories (C, D and E) involve more conventional agro-livestock systems providing predictable income flows, collateral and with low rates of default (up to 2%). Credit for poorer farmers on agrarian reform settlements and for special groups such as youth and women (categories A, B and A/C) is heavily subsidized (40%) and carries default rates of over 30%.

Bank managers are reluctant to release funds for *Proambiente*-type activities, especially for the typically poorer farmers on settlement schemes, since agroforestry and similar activities have traditionally been considered ‘unproductive’ due to their innovative and sometimes uncertain nature. They have a longer-term time horizon in which income flows can be unpredictable compared with normal farming systems. This induces extreme caution on the part of local bank officials, who tend to have little knowledge of these areas. It takes several years, for example, for planted trees to yield an income from fruit or timber sales, unlike with cattle or conventional food crops.^13^

‘Environmentally friendly’ credit lines have recently been introduced within PRONAF, but these are also unsuitable for most *Proambiente* activities. For example, 84% of ‘PRONAF-Forests’ funding is allocated to larger-scale reforestation schemes for Eucalyptus rather than agroforestry projects. ‘PRONAF-Agroecology’ requires detailed feasibility studies and is not suitable for small farmers in the absence of such technical assistance. ‘PRONAF-ECO’ is for water and energy projects, also granted on the basis of prior technical studies. Thus, one of the demands from extension workers and farmers involved in *Proambiente* is that MDA/PRONAF policies should be realigned with changing needs, a proposal that meets with significant internal resistance in the ministry itself.

Another related issue concerns the provision of agricultural extension support to *Proambiente* farmers. This is sub-contracted on an annual basis by the MDA to local non-governmental organizations. However, delays of several months in contract renewals have led to a lack of continuity in service provision, often at key points in the agricultural calendar, generating considerable frustration among NGOs and farmers. This has led to high rates of staff turnover (MMA 2004). Furthermore, state government extension services are rarely attuned to the *Proambiente* ethos and collaboration with their NGO counterparts seems to be an exception rather than the rule. In 2006, the MDA launched a new National Programme for Technical Assistance and Rural Extension (PRONATER), which aims to foster greater collaboration among federal, state and non-governmental extension services in support of small farmers generally, including those participating in *Proambiente* (MDA 2006, 2007).

Political bias in the identification of *Proambiente* ‘poles’ has also been raised as a problem that may have created obstacles to project implementation. Born of grassroots social movements and taken over by the PT government, a preference for areas characterized by traditions of rural trade unionism has been evident. While positive from a mobilization standpoint, this could distort the process by leading to the exclusion from *Proambiente* of organized but non-unionized communities. Furthermore, a unionized community does not necessarily have the organizational attributes or the social capital necessary to become successfully involved in a PES scheme. This is a key issue in the case of *Proambiente*, in which ‘community agreements’ form the basis for negotiating and mapping individual and group sustainable practices. Without a degree of group solidarity to ensure successful implementation, the ultimate objectives of certification and payments may be undermined.

Seen in more strategic terms, *Proambiente* was originally conceived as part of a longer-term process of territorial development in the Amazon in which support for small-scale farming would be integrated with other measures. Fears have been expressed that the failure to pursue such a coordinated approach with due prior attention being given to organizational and management capacity will frustrate the government's efforts to promote sustainable rural development adapted to regional conditions (MMA 2004; MDA 2005). Thus, the need for greater collaboration between the ministries of environment and agrarian development in the execution of *Proambiente* is evident.

## 5. Conclusion: better RED than dead

*Proambiente* is Brazil's incipient attempt to provide a system of PES rendered by small producers who are helping to conserve the Amazon rainforest by adopting more sustainable farming systems, including agroforestry, extractivism, forest and pasture management, among others. The need to provide compensation for RED is being debated under the UNFCCC following pressure from developing countries for avoided deforestation to be included as a valid criterion for carbon offsets under the CDM of the Kyoto Protocol after 2012. Such trading has already taken place in a small way through informal carbon markets (Bayon *et al*. 2006). Indeed, this path is being taken by the state of Amazonas, as noted above. Many people believe that further funding could be released, either through the market or via donor trust funds. The World Bank, for example, has set up a US$300 million FCPF to pilot RED schemes. Such initiatives could benefit thousands of poor family farmers in Amazonia and, indeed, the tropics generally, who depend for their livelihoods on natural resources but who struggle to make ends meet.

After just 4 years in operation and having achieved modest success, *Proambiente* has demonstrated pitfalls that threaten to frustrate the realization of this potential. As noted above, these include funding bottlenecks, limited implementation capacity, poor cross-sector coordination and possible political bias in planning. Very limited payments have been made to farmers on the basis of equally limited evidence of environmental service contributions. There has been almost no effective monitoring, quantification or certification of such services, nor systematic impact evaluation. Rather, it has worked on the basis of trust and a general, somewhat impressionistic, appreciation of the overall beneficial nature of participants’ activities. As such, *Proambiente* has been labelled as a ‘PES-like’ scheme that, in common with many other similar programmes, does not fulfil all the strict criteria involved in a formal PES exercise but which nevertheless has similar goals (Wunder 2006, p. 54).

There is another fundamental anomaly in terms of pursuing environmental versus social objectives. To conserve the greatest area of forest, large landowners should be targeted since in Amazonia at least they occupy most of the region and pose the most serious threat to the environment overall through illegal logging, cattle ranching and other commercial activities. Furthermore, the transaction costs of dealing with a small number of larger owners would be relatively low. In terms of social justice, however, smallholders and other poor resource users should be prioritized, although they are responsible for a relatively small percentage of deforestation and are expensive to reach. At the same time, care has to be taken to protect the rights of indigenous and traditional populations (Griffiths 2007). It has been argued that PES ‘are best suited to scenarios of moderate conservation opportunity costs on marginal lands and in settings with emerging, not realized threats’ (Wunder 2006, p. 48).

Yet in spite of widespread support among NGOs for PES generally, some members of the academic and scientific community in Brazil oppose it on the grounds that such funds are likely to be monopolized by large-scale farmers and small producers would be excluded (ESP 2007). Care would have to be taken to ensure that support is directed to those areas characterized by on-going tension over access to land and natural resources, which places serious pressure on the forest. There is much potential for targeting small farmers in precarious situations who could benefit from PES and be persuaded to modify their activities. One significant category involves land reform beneficiaries, whose settlement projects currently occupy 8% of Amazonia, but where the level of deforestation is four times the regional average. This is due to the fact that small farmers, lacking alternative, viable models or government support, continue with slash-and-burn agriculture and pasture formation as their major livelihood strategies (Epoca 2007).

Yet many small producers, including the bulk of those participating in *Proambiente*, while not exactly living in harmony with nature, are predisposed in some measure towards adopting more diversified, sustainable production systems if given the opportunity. They tend to appreciate the environmental and personal benefits that accrue, for example, to switching from slash-and-burn farming to agroforestry and extractive systems. They also understand that in the longer-term, the economic returns to perennials can be substantially larger than for traditional subsistence crops such as rice, beans and cassava. However, given the initial opportunity costs of setting up diversified farming systems, support is often needed to bridge the gap in constructing a new livelihood while these activities come on stream over a period of several years.

Through schemes such as *Proambiente* and other (non-carbon related) programmes that combat poverty and deforestation, the small farmers of Amazonia have shown that there are viable alternatives to the destructive practices that currently cause high rates of forest loss.^14^ However, adapting this potential specifically to reducing GHG emissions and scaling up to regional level will require much greater political commitment as well as the appropriate allocation of financial and human resources. Additional funding targeted at key groups of environmental custodians to support PES could serve to strengthen environmental policy and complement more traditional conservation measures. This is especially true for those producers living close to the economic margin whose behaviour could be modified through the provision of economic incentives and appropriate technical support.

Payments schemes such as *Proambiente* could eventually contribute significantly towards averting global warming, as long as this potential can be effectively harnessed. The possible integration in due course of *Proambiente* and other local–national PES initiatives into an international programme for promoting RED perhaps offers some cautious optimism for the future. Such a stance does not of course negate the potential problems of governance and management associated with such an ambitious global initiative, especially as far as Brazilian Amazonia is concerned and the sensitive political issues are involved. On balance, however, using PES to promote reduced emissions from deforestation could help avert the potentially fatal consequences of current development patterns in Amazonia upon both the environment and on people's livelihoods. In spite of the many challenges which must be faced, therefore, it is far better to be RED than dead.

## Endnotes

1 Fieldwork on *Proambiente* was carried out by the author in August 2007. Several project sites were visited in Tocantins and Acre, where interviews were conducted with farmers, local NGOs and state government officials. The author would like to express his gratitude to staff at Brazil's Ministries of the Environment and Agrarian Development for their valuable assistance, as well as to *Proambiente* project staff and beneficiaries interviewed during fieldwork. Special thanks are due to Shigeo Shiki, Hurben Corrêa da Silva and Brent Millikan for their support.

2 Although IBAMA refuses to publish figures on payment of fines, it is generally thought to be around 3–5%. The state government of Mato Grosso, for example, has made it known that just 2% of its environmental fines are collected (ISA 2006).

3 From 1971 to 1987 alone, for example, over US$5 billion in tax incentives and subsidized credit was allocated to cattle ranching in the Amazon (Schneider 1992). Although new incentives were suspended in 1991, already existing tax breaks as well as subsidized credit have continued (Fearnside 2000). From 1989 to 2002 the Bank of Amazonia lent US$5.8 billion to cattle ranchers in the region at subsidized rates (Arima *et al*. 2005).

4 The Rainforest Coalition is an inter-governmental organization involving over 30 countries, led by Papua New Guinea and Costa Rica, which are pledged to offering voluntary reductions in carbon emissions in exchange for access to international markets for emissions trading. In December 2005, the UNFCCC Parties agreed to consider the issue of RED (compensation for avoided deforestation) with a view to formulating recommendations to be discussed at the next Convention of the Parties (COP-13) held in December 2007 in Bali, Indonesia.

5 The Nova Gerar landfill gas scheme in Rio de Janeiro was the first project officially registered under the CDM. A small number of forestry projects based on reforestation of degraded areas have been undertaken in Minas Gerais (Plantar and Peugeot) and Mato Grosso (Bananal Island). See May *et al*. (2003) and Grieg-Gran *et al*. (2005). The Boticário Foundation has also announced plans to pay $12 million over 10 years to landowners who preserve the Atlantic Rainforest at the headwaters of Greater São Paulo's rivers (Nascimento Madureira 2006). This is comparable with schemes introduced elsewhere such as Colombia.

6 This process was initiated by rural trade union federations and major NGOs, such as IPAM and FASE, with financial support from the Ministry of the Environment, the State University of New York (SUNY) and the Ford Foundation.

7 Namely, Pará, Tocantins, Acre, Mato Grosso, Amapá, Roraima, Amazonas and Maranhão.

8 Lei de Mudanças Climáticas e Conservação Ambiental.

9 In addition to Amazonas, the states of Acre and Paraná are considering setting up state-led PES systems.

10 Projeto de Lei 792 (2007) introduced by Deputy Anselmo de Jesus (PT-Roraima) and Projeto de Lei 1190 (2007) authored by Deputy Antonio Palocci (PT-São Paulo).

11 The preliminary text of this third bill was discussed at the third meeting of the *Proambiente* Governing Council on 28–29 August 2007. It will probably be appended to the other two bills for the passage through Congress. Possible funding sources include fuel taxes (CIDE), the petrol and environment compensation fund, the Ecological VAT, the National Bank for Economic and Social Development (BNDES), regional development funds (FCO and FNO), as well external funding from aid and private sources (Arruda *et al*. 2007).

12 Observations in this section are based on interviews with staff at the Ministry of Agrarian Development, Brasilia, and conversations with Proambiente staff in the field, August 2007.

13 For example, one of the most popular agroforestry crops is cupuaçu (*Theobroma grandiflorum*), which requires 4–5 years to yield a viable first harvest. For valuable hardwoods such as mahogany, this period is measured in decades rather than years.

14 Other programmes include: the G7 Pilot Programme to Conserve the Brazilian Rainforest, the Plan to Combat Amazonian Deforestation, the National Pact for the Valorization of the Forest and the End of Deforestation in Amazonia and the 2-year moratorium agreed between NGOs and major agribusiness corporations on trading in soya bean produced in newly deforested areas (Hall 2000; Greenpeace 2007).
